# Understanding the genetics and epigenetics of bulimia nervosa/bulimia spectrum disorder and comorbid borderline personality disorder (BN/BSD-BPD): a systematic review

**DOI:** 10.1007/s40519-019-00688-7

**Published:** 2019-05-22

**Authors:** Sydney McDonald

**Affiliations:** Division of Medicine, University College London, Gower Street, London, WC1E 6BT UK

**Keywords:** Bulimia nervosa, Bulimia spectrum disorder, Borderline personality disorder, Genetics, Epigenetics

## Abstract

**Purpose:**

To evaluate and understand the genetic and epigenetic basis of bulimia nervosa/bulimia spectrum disorder and comorbid borderline personality disorder (BN/BSD-BPD).

**Methods:**

The present systematic review was conducted in accordance to PRISMA guidelines. Advanced systematic searches of Medline, EMBASE, PsychINFO, Web of Science, Scopus, CINHAL plus, and the Cochrane Library were conducted using the search terms ‘bulimia nervosa’, ‘bulimia spectrum disorder’, ‘borderline personality disorder’, ‘genes’, and ‘genetics’. The search strategy garnered seven studies for inclusion in the present review.

**Results:**

Women with BN/BSD-BPD had significantly lower serotonin and monoamine oxidise activity compared to women with BN/BSD or healthy controls (HC). As well, women with BN/BSD-BPD displayed elevated methylation of the dopamine receptor gene promoter, brain-derived neurotrophic factor, and changes in the methylation of the glucocorticoid receptor gene promoter (NR3C1) compared to women with BN/BSD and HC. The results also demonstrated that rates of childhood sexual abuse and childhood physical abuse are higher in those with BN/BSD-BPD than those with BN/BSD and HC, and that these types of abuse are often correlated with the methylation differences seen in BN/BSD-BPD women.

**Conclusion:**

Due to the differences observed between individuals with BN/BSD-BPD and those with BN/BSD and HC a genetic/epigenetic aetiological model of BN/BSD-BPD was developed and is proposed in this review. This evidence-based model visually illustrates the current state of the field and draws attention to the need for subsequent research.

**Electronic supplementary material:**

The online version of this article (10.1007/s40519-019-00688-7) contains supplementary material, which is available to authorized users.

## Introduction

### Bulimia nervosa, bulimia spectrum disorder, and borderline personality disorder

#### What is bulimia nervosa and bulimia spectrum disorder?

Bulimia nervosa (BN) is a multifaceted eating disorder (ED) characterized by an overvaluation of body shape and/or weight on self-image, coupled with recurrent episodes of binge eating and compensatory behaviours in attempts to lose weight [1]. The Diagnostic and Statistical Manual of Mental Disorders, fifth edition (DSM-5) includes both BN and bulimia spectrum disorder (BSD), however, BSD is referred to as “bulimia nervosa of low frequency and/or limited duration” [2]. Many studies include both BN and BSD individuals to represent the bulimic population as these groups do not differ considerably on most clinical indices [3]. The thoughts and behaviours of those with BN and BSD are the same; the only difference between the two groups is the frequency of which the individual engages in the bulimic behaviours. Specifically, the DSM-5 states that BN individuals engage in bingeing and compensatory behaviours at least once a week (on average) for a minimum of 3 months, whereas BSD individuals experience binge eating episodes and inappropriate compensatory behaviours less than once a week on average and/or for less than 3 months [2]. Thus, BSD can be defined as BN with limited frequency of compensatory behaviours. For simplicity and continuity, the present review refers to both these groups as BN/BSD.

#### What is borderline personality disorder?

Borderline personality disorder (BPD) is an intricate disorder characterized by affect instability, poor impulse control, impaired interpersonal relationships, and unstable self-image [4]. Complex comorbidity is a hallmark of BPD, and affected individuals demonstrate a greater likelihood of presenting with three or more psychiatric diagnoses compared to those without BPD.

#### Comorbidity of bulimia nervosa/bulimia spectrum disorders and borderline personality disorder

BPD presents alongside BN/BSD in approximately 35% of cases [5]. As these conditions are often comorbid they share many of the same risk factors, phenotypes, and behavioural traits including: attachment disturbances [6], adverse childhood experiences [7], non-suicidal self-injury [8], interpersonal difficulties [4, 9], and comorbid maladaptive mood and impulse regulation behaviours [10, 11]. In lieu of the large degree to which BPD interferes with interpersonal functioning, sense of self, and quality of life, it can be hypothesized that the 35% of those with BN/BSD and comorbid BPD (BN/BSD-BPD) face unique challenges. As well, it is believed that BN/BSD and BPD behaviour interact to hinder treatment as irregular eating patterns seem to intensify dysregulated moods, impulsivity, and other maladaptive symptomology, which, in turn, perpetuate the disordered eating behaviour as a coping strategy [12]. Little research has focused on the 35% of BN/BSD sufferers with comorbid BPD. As this condition is understudied and sufferers are greatly impacted by the disorder, research focused on understanding the genetic and epigenetic changes pertinent to BN/BSD-BPD will likely benefit the care given to these individuals, increase sufferer’s quality of life, and lead to better recovery outcomes for this population as efforts can be made to specifically target the individual’s biological changes and associated behaviours and symptoms.

### Biological variables associated with both bulimia nervosa/bulimia spectrum disorder and borderline personality disorder

In addition to shared behavioural traits, individuals with BN/BSD-BPD are also similar on many genetic and epigenetic variables. This will be discussed below and their prevalence will be investigated in the subsequent research. It is important to note that genome-wide association studies reveal connections between the genes relevant to the current research and other psychopathologies; therefore, they are not unique to BN/BSD and BDP symptomology [13]. This does not negate the importance of the genetics/epigenetics relevant to BN/BSD-BPD, it is simply important to be aware of the boarder context of the research. Furthermore, it is important to note that it is not known for certain whether these genetic/epigenetic changes are premorbid or if they arise due to BN/BSD-BPD behaviours. However, understanding the genetic variants associated with the behaviours and symptoms of BN/BSD-BPD may help explain an individual’s response to treatment and could help improve treatment outcomes in this population [14]. Previous research has found that those with BN/BSD-BPD experience more severe psychopathology, reduced global psychosocial functioning, increased interpersonal difficulties, and more frequent hospitalizations compared to BN/BSD individuals without comorbid BPD [15]. Furthermore, psychosocial treatment is thought to be less effective in individuals with BN/BSD-BPD compared to those with BN/BSD [16], and BN/BSD-BPD is associated with an increased risk of negative long-term outcomes compared to BN/BSD [17]. Understanding the genetic/epigenetic characteristics of BN/BSD-BPD may explain this observed variance in treatment response and could be used to further refine treatment for the individual.

#### Serotonin

It is widely established that central serotonin and eating behavior interact. Both human and animal studies have demonstrated an inverse relationship between serotonin neurotransmitter activity and food consumption [18]. Further, when single photon emission computer tomography was employed to compare serotonin transporter levels in women with BN/BSD and age-matched healthy controls a significant negative correlation between serotonin transporter levels and illness duration in the BN/BSD population was observed (*p* = 0.042, *r* = − 0.65), indicating that those with BN/BSD have less serotonin transporter availability than healthy counterparts [19]. Taken together this suggests decreased serotonin activity corresponds with BN/BSD.

The serotonin system has also been implicated in mood regulation, social behaviour, and impulsivity—all characteristics of BPD [20]. Other studies have implemented central serotonin in BPD, particularly in relation to chronic feelings of emptiness [21], impulsiveness [22], suicidality, and emotional dysregulation [23]. Thus, decreased central serotonin has been implicated in both BN/BSD and BPD.

The 5-hydroxytryptamine [5-HT] transporter polymorphism (5-HTTLPR) has been correlated with low transcription of the serotonin transporter protein, increased depression after tryptophan depletion (the precursor to serotonin), and clinical manifestations of both BN/BSD and BPD (discussed above) [24]. Polymorphisms of this transporter gene in relation to BN/BSD and BPD will be investigated further in the present systematic review.

#### Monoamine oxidase

Monoamine oxidase (MAO) is an enzyme involved in removing the neurotransmitters serotonin, dopamine, norepinephrine, and epinephrine from the brain [25]. Many studies have used platelet MAO activity as an index of these neurotransmitters, and have used blood levels of these neurotransmitters as a proxy for MAO activity in individuals with BN/BSD and BPD. Lower MAO activity is often observed in individuals with BN/BSD compared to healthy controls [26–28], however, decreased MAO activity is also sometimes seen in AN-R patients and, therefore, cannot be exclusively attributed to BN/BSD [28].

Low MAO activity has also been correlated to BPD symptoms including impulsiveness, affect dysregulation, sensation seeking, substance abuse, and suicide ideation and attempts [29, 30]. As well, decreased platelet MAO activity has been implicated in cluster B personality disorders, including BPD [31]. As low MAO activity has been implicated in both BN/BSD and BPD, this review will examine its potential role in BN/BSD-BPD.

#### Epigenetics

Epigenetic changes occur when a hereditary susceptibility is activated because of environmental pressures [32]. One common epigenetic mechanism is DNA methylation. DNA methylation occurs when methyl groups are added to the cytosine of CpG islands (frequently found in the regulatory region of most genes) and leads to reduced DNA accessibility, decreased transcriptional activity, and inhibition of gene expression [33, 34]. DNA methylation is not the only epigenetic modification that occurs in humans, other common epigenetic mechanisms include histone modification and noncoding RNA action [35]. The details of these epigenetic regulation mechanisms are beyond the scope of the present systematic review as all of the epigenetic studies included in the review only report epigenetic regulation resulting from DNA methylation; however, it is important to be aware that DNA methylation is not the sole epigenetic change that occurs. Childhood abuse is frequently cited as the “environmental pressure” that is associated with epigenetic effects correlated with BN/BSD and BPD symptomology and behaviour [32, 33]. Many other factors beyond abuse affect epigenetic regulation, some of these factors include: environmental pollutants, nutritional factors, and maternal behaviour (e.g., nursing behaviour or depression) [36]. Thus, although the epigenetic studies included in this review specifically examine the role of childhood abuse on the observed epigenetic changes, other factors also likely impact the epigenetic patterns observed. Furthermore, when interpreting the results presented in this review it should be noted that variability can exist between methylation levels of CpG sites within the same CpG island and this in turn may elicit differential gene expression [37]. Overall, epigenetic effects are complex and it is nearly impossible to attribute a change to a specific environmental factor, however, correlation between environmental factor (e.g., abuse) and epigenetic changes (e.g., DNA methylation) can be established. The present review will examine methylation of the glucocorticoid receptor promoter gene (NR3C1), brain derived neurotrophic factor (BDFN), and the dopamine receptor gene promoter (DRD2) in women with BN/BSD-BPD.

The glucocorticoid receptor is central to modulating individuals stress reactivity, and anomalies in this system have been associated with BN/BSD and BPD. Studies have associated BN/BSD with reduced inhibition of the hypothalamic–pituitary–adrenal stress response (a glucocorticoid receptor modulated response) [32]. As well, hyper-methylation of neuro-regulatory genes, including NR3C1, has been correlated with psychopathologies including suicidality, BPD, and BN/BSD [38]. Furthermore, childhood abuse has been associated with increased methylation of various glucocorticoid receptor gene exon-1 promoter variants in human studies [39]. As such, childhood abuse, methylation of the NR3C1, and BN/BSD-BPD may be correlated; this will be examined further in this review.

BDFN has been implicated in the regulation of food intake and energy homeostasis in the general population, and is observed to be reduced in AN and BN/BSD populations [33]. Studies have found that methylation of the BDFN gene differs between BPD and healthy control populations, with increased methylation seen in the BPD population [38]. As well, early developmental stress has been implicated in the hyper-methylation of the BDFN gene [40], again creating a potential connection between childhood abuse, BN/BSD, and BPD.

Finally, studies have found associations between the DRD2 Taq1 polymorphism and symptoms related to both BN/BSD and BPD (e.g., binge eating, emotional eating, food cravings, impulsivity, and novelty seeking) [41]. As with the previous two genes, childhood abuse is thought to be associated with methylation of the DRD2 gene [42], again indicating a potential link between childhood abuse, BN/BSD, and BPD.

### The scope of the current paper

The relationship between BN/BSD and comorbid BPD is complex and remains to be fully understood. The present paper will use current literature to explore the ways in which BN/BSD-BPD may be influenced by genetic and epigenetic factors. This paper will clearly outline these developments and will provide a coherent summary of the current state of the field. As well, a genetic/epigenetic aetiological model of BN/BSD-BPD will be proposed which summarizes the current findings and illustrates the need for future research.

## Methods

### Search strategy

An extensive electronic search was conducted to ascertain relevant papers for the current systematic review from the following databases: Medline, EMBASE, PsychINFO, Web of Science, Scopus, CINHAL plus, and the Cochrane Library (Table 1). Advanced searches were completed in all the aforementioned databases using the search terms ‘bulimia nervosa’, ‘bulimia’, ‘borderline personality disorder’, ‘borderline adj4 personality disorder’, ‘genes’, and ‘genetics’. The search was limited to articles containing one relevant term to each of the three categories of the search [1] bulimia nervosa, [2] borderline personality disorder, and [3] genetics (refer to online appendix 1 for a summary of the search strategy). No restrictions were placed on the year of publication, or the language.

**Table 1:** The number of articles gathered using the search strategy in November 2018, and the corresponding database

Database	Number of articles
CINAHL plus	4
Cochrane Library	5
EMBASE	24
Medline	10
PsychINFO	19
Scopus	983
Web of Science	21
Total	1066

#### Selection of studies

**Fig. 1:**
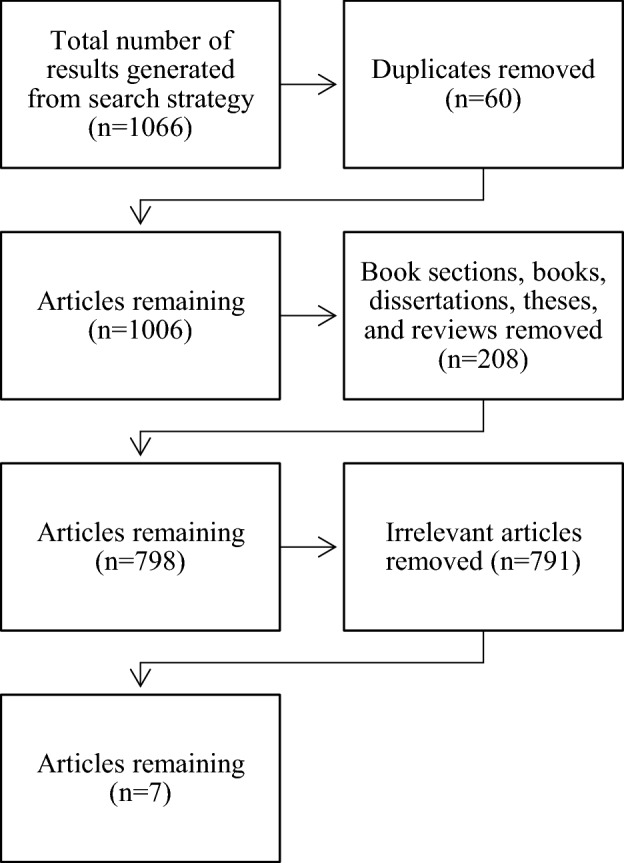
Search strategy process

The search strategy garnered a total of 1066 papers. After deduplication 1006 remained. Reviews, book sections, commentaries, theses and dissertations were then removed (*n* = 188). The articles were then reviewed to ensure they encompassed all three aspects of the inclusion criteria (bulimia nervosa/bulimia spectrum disorder, borderline personality disorder, and a genetic or epigenetic variable). This resulted in seven relevant articles to be included in the present review (Fig. 1).

### Procedure

To investigate the role of genetics and epigenetics in BN/BSD with comorbid BPD (BN/BSD-BPD) the articles identified through the search strategy were reviewed and grouped by topic. The results from each topic were summarized and discrepancies between studies were identified. The limitations of the studies were addressed and areas that require further research were outlined. Finally, the implications of better understating the ways in which genetics/epigenetics are involved in BN/BSD-BPD were discussed.

## Results

### Overview of studies

Seven studies fulfilled the inclusion criteria and were included in the review. These seven studies can be roughly classified into three groups: (1) polymorphisms of the serotonin transporter promoter (5-HTTLPR) gene (*n* = 2) [20, 24], (2) gene methylation of: the dopamine gene receptor (dopamine D2 receptor (DRD2) promoter (*n* = 1) [41], the glucocorticoid receptor gene (NR3C1) promoter) (*n* = 1) [32] and brain-derived neurotrophic factor (BDNF) (*n* = 1) [33], and (3) monoamine oxidize (MAO) activity (*n* = 2) [28, 43].

### Study characteristics

Most of the studies were conducted in Canada (*n* = 5), and the remaining studies were conducted in Spain (*n* = 2). Each study included in this review obtained informed consent from participants and were of cross-sectional design. The majority of the studies were independent from each other and recruited participants through ED services and advertisements in newspapers and university bulletins (*n* = 5), however, two studies obtained their samples from the same pre-existing database developed for a large-scale, institutional ethics board approved study.

The sample sizes of the studies included in the systematic review range from 59 to 308 (mean *n* = 126.67 SD = ± 90.31), with a total of 760 participants (note: this reflects the fact that two studies used the same sample of participants). All participants in the studies were female. Most of the studies included an ED group(s) and a healthy control group (*n* = 5), but two just involved individuals with an ED. Of the total sample size, 568 individuals were diagnosed with an ED and the remaining 192 participants were HC without an ED. ED diagnoses were based on the criteria outlined in the DSM-IV in six of the studies, however, the most recent study, conducted in 2014, used the DSM-5 criteria. Of the 568 who fit the criteria for an ED 506 were diagnosed with BN or BSD (89.1%), 19 with binge/purge type AN (AN-BP) (3.3%), 25 with restrictive AN (AN-R) (4.4%) and 18 with EDNOS (3.2%). BSD and/or BN were frequently used in the studies included in the present systematic review to encompass bulimia. The participants were 17 years to 49 years old, with the mean age ranging from 20.06 years to 26.05 years across studies.

The study aims varied between the seven studies; however, they all generally examined the relationship between BN/BSD and BPD along with a biological variable. Two studies explored the effects of polymorphisms in the 5-HTTLPR serotonin transporter promoter gene in individuals with BN/BSD-BPD; however, each study examined this relationship in the context of different additional variables. These variables include childhood abuse (sexual and physical) (*n* = 1) [20], impulsive behaviours (*n* = 2) [20, 24], insecure attachment (*n* = 1) [20], interpersonal difficulties (*n* = 1) [24], and affect instability (*n* = 1) [24]. One study also explored the 5-HTTLPR polymorphism in relation to the density of paroxetine-binding sites [24].

MAO activity in individuals with BN/BSD, BN/BSD-BPD, and HC was central to two studies [28, 43]. One study investigated differences in psychological traits (e.g., impulsivity) and MAO activity, as well as interactions between these variables in the aforementioned groups [43]. The second study had a similar objective to the first. This study aimed to understand the relationship between platelet MAO activity and borderline personality traits in individuals with an ED [BN/BSD, AN-BP, and anorexia nervosa not otherwise specified (AN-NOS)] and individuals with BN/BSD-BPD, compared to HC [28].

Three studies focused on the methylation of particular genes in women with BN/BSD-BPD, BN/BSD, and HC and associations with childhood abuse (sexual or physical) [32, 33, 41]. The genes under question were: brain-derived neurotrophic factor (BDNF) [33], the glucocorticoid receptor gene promoter (NR3C1) [32], and the dopamine receptor gene promoter (DRD2) [41]. When interpreting the results, it is important to note that DNA methylation was measured from DNA samples obtained from blood. As DNA methylation is cell-specific, the methylation patterns reported in the studies do not necessarily reflect DNA methylation of these genes in the brain.

The majority of the studies (*n* = 5) [20, 24, 32, 33, 41] employed the eating disorders examination (EDE) to identify if participants and controls had an ED or not [44]. Two studies did not use the EDE to identify ED in participants [28, 43], but in both these cases the Bulimic Investigatory Test Edinburgh (BITE) was commissioned [45]. Supplementary ED diagnostic tests were undertaken in three studies (refer to Table 2 for a full list) [20, 24, 28].

**Table 2:** Diagnostic tests employed by the studies

Diagnostic test	No. of studies (*n*)
Psychological measurements	
Bulimic Investigatory Test Edinburgh (BITE)	2
Eating Attitudes Test–26 (EAT-26)	2
Eating Disorders Examination (EDE)	5
Eating Disorders Inventory (EDI)	1
Structured Clinical Interview for DSM-IV Axis I Disorders (SCID-I)	3
Structured Interview for DSM-IV Axis II Disorders (SCID-II)	3
Diagnostic Interview Schedule, Version IV (DIS4)	2
Dimensional Assessment for Personality Pathology (DAPP)	2
Diagnostic Interview for Borderline Personality Disorder (DIB-R)	1
Zanarini Rating Scale (ZAN-BPD) for severity of BPD	1
Beck Depression Inventory (BDI)	1
Centre for Epidemiological Studies Depression Scale (CES-D)	1
Self-defeating personality subscale of the Millon Clinical Multiaxial Inventory (MCMI-II)	1
Barrat Impulsiveness Scale	3
Impulsivity Behavioural Scale-Revised (IBS-R)	1
Childhood Trauma Interview (CTI)	4
Clinician-Administered PTSD Scale (CAPS)	2
Computerized go/no go discrimination test to indicate disinhibition	1
Biological measurements	
Platelet MAO activity measured by isotopic methods	1
DNA extracted from blood and used for methylation analyzes by pyrosequencing methods	1
Urinary 24-h excretion of noradrenalin (NE), serotonin (5-HT), and dopamine (DA) and their main metabolites quantified using column chromatographic methods	1
Serum cortisol determined from a sample of fasted (12 h) blood	1
DNA methylation assessed using the EpiTYPER platform	2

In addition to the psychological tests, each study undertook biological measurements to identify the genetic or epigenetic variable under question. To perform the methylation analyses one study employed pyrosequencing techniques [41], and two used Epityper methods [32, 33]. Other, more specific procedures were done in most studies to properly investigate methylation of promoter genes, MAO activity, and polymorphisms of the 5-HTTLPR promoter gene (see Table 2 for more details).

### Key findings

Five of the studies compared ED groups to HC [28, 32, 33, 41, 43], while the remaining two studies simply focused on an ED population [20, 24]. All seven studies focused on BN/BSD as the ED under question. A few studies also looked at differences between other subtypes of EDs (*n* = 3), for example, AN-R and AN-BP [20, 24, 28]. Of the three studies that included various types of ED, one examined the rate at which BPD presented alongside each ED [28]. This study found that comorbid BPD was significantly higher in individuals with BN/BSD (33%), and AN-BP individuals (29.4%) than AN-R (12%). Additionally, covariates that could alter statistical significance of the findings were investigated in seven of the studies. These covariates were: frequency of binge and purge behaviour, BMI, and use of psychoactive medication. All seven studies found that these covariates did not alter the statistical significance of analyses, however, two studies used an un-medicated sample [28, 32]; therefore, the medication covariate was only relevant in five of the studies. Table 3 provides a summary of the studies included in the present review.

**Table 3:** Overview of the studies included in the present review

Study	Country	Sample characteristics	Aims	Diagnostic tools and measurements	Key findings
Díaz-Marsá et al. [28]	Spain	AN-R (25) AN-BP (14) AN-NOS (3) BN-P (29) BN-non-P (1) HC (28) Mean age of ED group: 25.6 years Mean age of control group: 24.4 years Mean BMI of ED group: 18.5 kg/m^2^ Mean BMI of control group: 20.7 kg.m^2^	To determine if MAO activity is correlated with ED and their comorbid personality disorders	SCID-II ZAN-BPD EDI BITE BIS11 Platelet MAO activity measured by isotopic methods	Comorbid BPD was significantly higher (*p* < 0.05) in BN (33%) and AN-BP (29.4%) than in AN-R (12%) ED-BPD patients had significantly lower MAO activity than ED no-BPD MAO activity was inversely correlated to measures related to impulsivity and severity of bulimic symptoms MAO activity was significantly lower in BN-BPD and AN-BP-BPD than in BN, AN-BP, and HC
Steiger et al. [32]	Canada	BSD (153) HC (102) Mean age of ED group: 24.7 years Mean age of control group: 23.7 years Mean BMI of ED group: 22.8 kg/m^2^ Mean BMI of control group: 22.4 kg/m^2^	To replicate previous findings that suggest methylation of the DRD2 promotor region is increased in women with BSD compared to HC To investigate associations between DRD2 methylation and (1) past childhood abuse (2) comorbid BPD	SCID-II EDE CTI DNA extracted and used for methylation analyzes by pyrosequencing methods	BSD-BPD group had significantly higher methylation of the DRD2 promoter than both the BSD and HC groups Women with BSD and a history of childhood sexual abuse had a significantly higher methylation of the DRD2 promoter than HC, and a marginally higher methylation of the DRD2 promoter than BSD-no childhood sexual abuse group No methylation differences of the DRD2 promoter was seen between the HC group and the BSD group
Steiger et al. [24]	Canada	BSD-P (37) BSD-non-P (3) AN-BP (4) EDNOS (15) Mean age: 25.5 years Mean BMI: 21.6 kg/m^2^	To investigate the implications of 5-HTTLPR polymorphisms for (1) eating symptoms, (2) psychological traits, and (3) platelet (^3^H) paroxetine-binding in women with BN	EDE EAT-26 BIS11 CES-D DAPP SCID-II Computerized go/no go discrimination test to indicate disinhibition DNA extracted and amplified by PCR	No differences were seen between the S/S, S/L, or L/L allelic variations of the 5-HTTLPR gene on eating symptoms The S allele of the 5-HTTLPR polymorphism was associated with significantly higher rates of affect instability, insecure attachment, command errors on the go/no go task, and with significantly lower density of the (^3^H) paroxetine-binding sites The S allele was correlated with a higher frequency of BPD (individuals meeting BPD criteria always had an S allele)
Steiger et al. [20]	Canada	BSD-P (70) BSD-non-P (4) BSD-NOS (18) Mean age: 25.2 years Mean BMI: 22.4 kg/m^2^	To examine the relationship between “dysregulation” in women with BSD, and a 5-HTTLPR polymorphism, and past sexual or physical abuse	EDE EAT-26 DAPP-BQ BIS11 SCID-II CTI DNA extracted and amplified by PCR	Using the biallelic classification of the 5-HTTLPR results indicated pathological elevations in stimulus seeking and insecure attachment in S-allele carriers who had experienced childhood abuse The S-allele was found to be a significant predictor of BPD Childhood abuse was found to be a significant predictor of BPD Results from a triallelic model indicated that combined S and L_G_ alleles predicted BPD at a trend level, and childhood abuse was a significant predictor of BPD
Steiger et al. [32]	Canada	BN-childhood abuse (32) BN-no childhood abuse (32) HC-no childhood abuse (32) Mean age of ED group: 26.1 years Mean age of control group: 23.4 years Mean BMI of ED group: 22.4 kg/m^2^ Mean BMI of control group: 21.8 kg/m^2^	To compare methylation levels of the glucocorticoid receptor (GR) gene (NR3C1) promoter in women with BN and HC To examine the extent to which methylation of NR3C1 corresponds to childhood abuse, suicidality, or BPD in women with BN	EDE SCID-I DIS4 CAPS CTI DNA extracted, amplified by PCR, and treated with sodium bisulphite DNA methylation assessed using the EpiTYPER platform	Elevated methylation on the GR exon site 1C was found in individuals with BN-BPD compared to levels observed in HC No differences in methylation levels were observed between BN and HC groups Lower methylation on the GR exon in the H1 region was observed in individuals with BN-BPD compared to HC Significantly elevated methylation on the GR exon in the 1C region was seen in individuals with BN-suicidal group compared to HC Methylation levels were not different between the BN-suicidal group and the HC group or between the BN-suicidal and BN-non-suicidal groups
Thaler et al. [33]	Canada	BN-childhood abuse (32) BN-no childhood abuse (32) HC-no childhood abuse (32) Mean age of ED group: 26.1 years Mean age of control group: 23.7 years Mean BMI of ED group: 22.4 kg/m^2^ Mean BMI of control group: 21.8 kg/m^2^	To examine the degree to which brain-derived neurotrophic factor (BDNF) methylation corresponds to (1) bulimic or normal-eating status, (2) a history childhood abuse, and (3) comorbid BPD	EDE SCID-I DIS4 CAPS CTI DNA extracted, amplified by PCR, and treated with sodium bisulphite DNA methylation assessed using the EpiTYPER platform	Greater methylation on chromosome 11: 27,722,840-27,723,980 was observed in both groups of BN participants compared to HC Individuals with BN and physical abuse had greater methylation than controls Individuals with BN but without physical abuse had greater methylation than controls Individuals with BN and sexual abuse had greater methylation than controls Individuals with BN but without sexual abuse had greater methylation than controls Individuals with BN-BPD had greater methylation than controls Opposite methylation patterns were observed compared to other regions on chromosome 11: 27,723,290. Here controls displayed greater methylation than did those with BN, BN with and without physical abuse, BN with no sexual abuse, and BN no-BPD
Vaz-Leal et al. [43]	Spain	BN-P (75) HC (30) Mean age of ED group: 22.9 years Mean age of control group: 23.6 years Mean BMI of ED group: 22.5 kg/m^2^ Mean BMI of control group: 22.0 kg/m^2^	To analyse the relationship between a set of neurological variables (hypothalamic-pituitary adrenal axis and MOA activity) and a set of psychopathological variables in individuals with BN and in HC	BITE BDI IBS-R MCMI-II DIB-R Serum cortisol determined from a sample of fasted (12 h) blood Urinary 24-h excretion of noradrenalin (NE), serotonin (5-HT), and dopamine (DA) and their main metabolites were quantified using column chromatographic methods	Women with BN displayed significantly higher scores on all psychopathological variables, had a lower 24-h urinary excretion of 5-HT and DA, and a lower cortisol suppression rate after DXT administration compared to HC In the BN group, low cortisol suppression rates in combination with either low DA or its metabolite HBA were found to be predictive of psychopathological behaviour that is partially associated with elevated 5-HT levels

Two studies examined the 5-HTTLPR polymorphism in women with BN/BSD-BPD. Traditionally, the 5-HTTLPR polymorphism was categorized using a biallelic model which included an S (short) allele and an L (long) allele, which coded for low or high serotonin transporter activity, respectively [46]. However, more recent research argues for a triallelic model of the 5-HTTLPR polymorphism as evidence suggests there is a “low-frequency” L allele variant, L_G_ which functions like the low-function S allele. This variant is simply an L allele containing an A → G single-nucleoid polymorphism in its sequence. Simply, the S and L_G_ alleles can be classified as “low-function” variants and the L_A_ allele can be classified as a “high-function” variant [47]. One of the studies conducted analyses using both the biallelic and triallelic models [20], and the other study used only the biallelic model [24] (see Table 4 for genotype allele distribution of samples). Neither study found an association between allelic variants and eating pathology (e.g., binge and purge frequency, BMI, and one found no association between allelic variants and body dissatisfaction or eating attitudes (however, one study did not look at this). Both studies analysed the relationship between BPD and allele frequency and both found that those who met the criteria of BPD had higher frequencies of the S allele than their non-BPD counterparts. All the individuals with BPD were found to be carriers of the S allele (*n* = 1) [24], and in another study 90.5% of individuals with BPD were carriers of the S allele [20]. In comparison, only 50.8% of individuals without BPD were carriers of the S allele (*n* = 1) [20]. When the biallelic model was used in analyses, the S allele was associated with significantly higher instability [*t*_(53)_ = − 2.46, *p* < 0.02, *d *= 0.71] (*n* = 1) [24], insecure attachment [*t*_(53)_ = − 2.43, *p* < 0.02, *d *= 0.70] (*n* = 1) [24], affect instability [*t*_(53)_ = − 2.46, *p* < 0.02, *d *= 0.71] (*n* = 1) [24], disinhibition [*t*_(53)_ = − 2.23, *p* < 0.03, *d *= 2.98] (*n* = 1) [24], and a significantly lower density of [^3^H-] paroxetine-binding sites [*t*_(53)_ = 2.38, *p* < 0.03, *d *= 0.64] (*n* = 1) [24]. When relationships were analysed using the triallelic model no significant effects were obtained in relation to stimulus seeking and insecure attachment, but the results pointed in the same direction as the biallelic model (*n* = 1) [20].

**Table 4:** Genotype allele distributions of samples (% of sample size)

Study	Biallelic model			Triallelic model		
	S/S (low–low) (%)	S/L (low–high) (%)	L/L (high–high) (%)	S/S, S/L_G_, L_G_/L_G_ (low–low) (%)	L_G_/L_A_, S/L_A_ (low–high) (%)	L_A_/L_A_ (high–high) (%)
Steiger et al. [24]	22.0	47.5	30.5	–	–	–
Steiger et al. [20]	19.6	46.7	33.7	31.5	44.6	23.9

Two studies examined MAO in relation to groups of women with BN/BSD-BPD, BN/BSD, and HC [28, 43]. One focused on platelet MAO activity measured through isotopic measures [28], whereas the other focused on clinical variables that reflect MAO activity determined through 24-h urinary excretion [43]. These clinical variables included: serotonin, dopamine, norepinephrine, as well as their metabolites (5-hydroxyindole-acetic acid, homovanillic acid, and 3-methoxy-4-hydroxyphenylglycol, respectively).

The study that examined platelet MAO found that ED patients had significantly lower MAO activity than HC (*p* < 0.01, *d *= 0.76) [28]. As well, MAO activity was markedly lower in BN/BSD and AN-BP patients with comorbid BPD than ED counterparts without BPD (*t *= 4.7, *p* < 0.05, *d *= 0.63). Furthermore, this study observed that platelet MAO activity was inversely related to the severity of BN/BSD symptoms (*r* = − 0.31, *p* < 0.05) as well as the severity of BPD (*r* = −0.42, *p* < 0.01).

Clinical variables that reflect MAO activity were significantly lower in BN/BSD patients than HC (24-h excretion of serotonin [RoM = 0.7 (0.5–0.9), *p* < 0.01]; 24-h excretion of dopamine [RoM = 0.8 (0.7–0.1), *p* < 0.04] [43]. Furthermore, MAO activity of BN/BSD-BPD patients was significantly lower than BN/BSD patients (borderline personality traits x dopamine excretion (95% CI − 0.85 to − 0.09, *p* < 0.02). This relationship was not significant in the HC group. Additionally, this study observed the cortisol suppression rates in relation to BN/BSD and BPD. The findings parallel those previously stated, with BN/BSD patients displaying a significantly lower cortisol suppression rate than HC [RoM = 0.9 (0.9–0.8), *p* < 0.002], and BN/BSD-BPD patients having a lower cortisol suppression rate than BN/BSD patients (95% CI − 5.88 to − 2.93, *p* < 0.001).

One study focused on methylation of the dopamine receptor (DRD2) promoter gene located on chromosome 11q in women with BN/BSD-BPD [41]. It was observed that women with BN/BSD and HC do not differ in mean methylation levels of the DRD2 (mean methylation = 7.35% ± 0.67 and mean methylation = 7.11% ± 0.72, respectively). However, women with BN/BSD-BPD showed significantly higher levels of DRD2 methylation compared to HC (*p* < 0.05, *d *= 0.90), and slightly higher levels of DRD2 methylation compared to women with BN/BSD (*p* < 0.10, *d *= 0.76).

The methylation of the glucocorticoid receptor gene promoter (NR3C1) exon sites 1B, 1C, 1F, and 1H in women with BN/BSD-BPD, BN/BSD, and HC was explored in one study [32]. ANOVA analyses indicated that a significant group × promoter-region site interaction in the 1C region was observed when testing BN/BSD-BPD vs. BN/BSD vs. HC groups [*F*(34,246) = 1.75, *p* < 0.02]. When looking at methylation levels in the 1C region it was observed that the BN/BSD-BPD group had elevated methylation compared to methylation levels of HC (*p* < 0.03). When comparing methylation levels between BN/BSD and the HC groups no differences were observed. Methylation differences at the 1H region were also observed. Here, the BN/BSD-BPD group exhibited significantly lower methylation than the HC group [*F*(2,88) = 4.86, *p* < 0.01]. Once again, methylation levels in the BN/BSD did not differ from controls.

Finally, no significant methylation differences were observed between BN/BSD individuals and HC. However, a nonsignificant trend towards a group × promoter-region site interaction was observed between these two groups [*F*(17,78) = 1.54, *p* < 0.10]. This is consistent with previous findings of the hyper-methylation of some sites in the 1C region in the BN/BSD groups compared to the HC group.

One study examined methylation of brain-derived neurotrophic factor (BDNF) in women with BN/BSD-BPD, BN/BSD, and HC [33]. Methylation patterns were examined at the BDNF promoter-region fragment, located on chromosome 11: 27,722,840–27,723,980. When methylation patterns were analysed between the BN/BSD and the HC groups significant methylation patterns were observed and BN/BSD individuals displayed elevated methylation compared to HC across various CpG regions (ERR = 1.45, 95% CI 1.07, 1.98, *p* values ranging from 0.003 to 0.036). Similarly, when comparing methylation between individuals with BN/BSD-BPD to HC the BN/BSD-BPD group showed significantly higher methylation levels across various CpG sites (ERR = 2.87, 95% CI 1.57, 5.54, *p* values ranging from 0.01 to 0.013).

The effects of childhood abuse (physical and sexual) on the relationship between BN/BSD, BPD, and a biological variable were investigated in four studies. These studies included the genetic and epigenetic variables: the 5-HTTLPR polymorphism (*n* = 1) [20], methylation of the DRD2 receptor gene (*n* = 1) [41], methylation of NR3C1 promoter gene (*n* = 1) [32], and methylation of BDNF (*n* = 1) [33].

When examining childhood abuse (physical and sexual) in relation to BN/BSD-BPD and the 5-HTTLPR polymorphism, childhood abuse had a significant effect on the relationship between BN/BSD and BPD using both the biallelic model and the triallelic model of the 5-HTTLPR (OR 3.27, 95% CI 1.09–9.18, *p* < 0.04 and OR 2.92, 95% CI 1.02–8.37, *p* < 0.05, respectively) [20]. Interestingly, 57.1% of individuals with BPD had been abused in childhood and only 34.8% of those without BPD experienced childhood abuse.

The study that focused on methylation of the DRD2 receptor gene in women with BN/BSD and BPD looked at childhood sexual abuse and childhood physical abuse separately [41]. This study found a group effect associated with childhood sexual abuse [*F*(2,68) = 2.687, *p* < 0.075], but not childhood physical abuse in women with BN/BSD-BPD. Pairwise comparisons of mean methylation of the DRD2 receptor gene revealed significantly higher levels in women with BN/BSD and childhood sexual abuse compared to HC (*p* < 0.03, *d *= 0.84) and marginally higher methylation in women with BN/BSD and childhood sexual abuse compared to women with BN/BSD and no childhood sexual abuse (*p* < 0.07, *d *= 0.65).

The findings of the paper that looked that BN/BSD and BPD in relation to methylation of NR3C1 promoter were contrary to the findings of the previous two studies [32]. Here, no significant or main interaction between BN/BSD-abused women vs. BN/BSD non-abused women vs. HC when an ANOVA test was run.

Finally, the study looking at BN/BSD, BPD, and BDNF methylation in women found that women with BN/BSD and childhood physical abuse displayed greater methylation than HC across CpG sites on chromosome 11: 27,722,840–27,723,980 (*p* values ranging from 0.001 to 0.017, and main effect *p* = 0.03) [33]. When looking at childhood sexual abuse it was observed that women with BN/BSD and childhood sexual abuse had greater percentage methylation than HC (ERR = 1.55, 95% CI 1.08, 2.21, *p* = 0.017).

### Limitations of the studies

Here the limitations of the seven studies included in the systematic review will be discussed. Table 5 outlines the limitations that pertain directly to each study. The demographics of the studies create some limitations. Firstly, the sample of each study was completely female, leading to results biased by gender. That being said, the prevalence of BN/BSD is higher in females than males with a ratio of 10:1 [48] and there is some evidence to suggest that females are more affected by BPD than males [4]. Therefore, the results will be specific to females with BN/BSD-BPD and could inform treatment and prevention in this population more effectively than research done on both males and females. All of the studies were conducted in Western countries, five in Canada and two in Spain. This creates a westernized prejudice in the conclusions and limits the degree to which the results can be extrapolated to diverse populations and ethnicities. Furthermore, research suggests that affects epigenetic expression; for example, diet, lifestyle, maternal care, and exposure to environmental toxins are all factors that affect DNA methylation patterns [49]. As Canada and Spain are both developed countries they share many environmental factors that would likely be different in developing countries. This further limits the generalizability of the studies included in the present review as many focus on epigenetics, and the epigenetic changes discussed could be influenced by the permissive culture found in both Canada and Spain. Additionally, the ages of the participants ranged from 17 to 49 years old, further narrowing the specificity and relevance of the findings.

**Table 5:** Limitations of the studies included in the systematic review

Study	Study limitations
Díaz-Marsá et al. [28]	Sample 100% female Small sample size (*n* = 100) Small sample size of ED groups is too small to allow comparisons of platelet MAO reduction between ED subtypes
Steiger et al. [32]	Sample 100% female Small sample size (*n* = 308) Small observed group differences in the mean methylation of the DRD2 promoter gene (~ 1%) Measures of DNA methylation obtained through peripheral biomarkers Lack of “neutral’ control gene Many confounding variables were not assessed or controlled for
Steiger et al. [24]	Sample 100% female Small sample size (*n* = 59) Discrepancies in findings when using the biallelic vs triallelic model of the 5-HTTLPR promoter gene Measures of DNA methylation obtained through peripheral biomarkers
Steiger et al. [20]	Sample 100% female Small sample size (*n* = 92) Discrepancies in findings when using the biallelic vs triallelic model of the 5-HTTLPR promoter gene Measures of DNA methylation obtained through peripheral biomarkers
Steiger et al. [32]	Sample 100% female Small sample size (*n* = 96) Measures of DNA methylation obtained through peripheral biomarkers Lack of “neutral’ control gene Treatment of medication use as a unitary effect, rather effect based on medication used No control for the confounding effect medication use may have on analyses
Thaler et al. [33]	Sample 100% female Small sample size (*n* = 96) Measures of DNA methylation obtained through peripheral biomarkers Lack of “neutral’ control gene Lack of control groups for the statistical analyses involving multiple comparisons
Vaz-Leal et al. [43]	Sample 100% female Small sample (*n* = 105) made comparisons between cortisol suppressors and cortisol non-suppressors impossible (non-suppressors in control group, *n* = 0; non-suppressors in ED group, *n* = 7) Use of 24-h urine excretion as the source of data for neurotransmitter activity Did not assess potential comorbid psychopathologies in the ED group

Each study had a small sample size (*n* = 59 to *n* = 308, mean *n* = 126.66 SD = ± 90.31), which hinders the conclusiveness of results through decreasing the confidence interval of the study, limiting the power of the study, and increasing the margins of error. This could prevent the existence of effect to be detected and leads researchers to question the clinical significance of the results. In some cases, the small sample size prevented researchers from analysing relationships of interest (see Table 5).

It is important to note that the seven studies that met the inclusion were all candidate gene studies. Although these studies do provide valuable information about the gene in question, they do not explain how the gene may influence others, nor do they provide a comprehensive understanding the genetic complexities of BN/BSD-BDP. As such, when interpreting the results from the present systematic review it must be noted that these genes are only a very small piece of the genetic/epigenetic aetiology of BN/BSD-BPD. Much more research is needed to more fully understand the genetic/epigenetic complexities of BN/BSD-BPD, including identifying additional genetic/epigenetic variables at play and determining the ways in which the genetics/epigenetics discussed in the present review influence other genes.

BPD and BN/BSD often present alongside many other psychopathologies (e.g., major depressive disorder, major affective disorder, and anxiety disorder) but none of the studies controlled for this potential covariate. Additionally, it would be impossible to control for all confounding variables, so many were left unaccounted for (e.g., nutrition and lifestyle).

It is also important to note that shared genetics/epigenetic variables as well as behavioural traits are observed in BN/BSD-BPD are not exclusive to this condition and are also observed in other psychiatric conditions. However, without more robust research regarding the genetics/epigenetic of psychological disorders it is difficult to fully understand the scope of influence the genetic/epigenetic variables examined in this study have on BN/BSD-BPD compared to other psychological conditions. As well, in relation to the genetic aspect of the studies, no “neutral” control genes could be included as they often do not exist. Therefore, it is impossible to be certain that the effects observed are the result of gene specific epigenetic results, rather than genome-wide effects.

Limitations associated with the use of both the biallelic and triallelic model of the 5-HTTLPR polymorphism exist as results differ depending on the model used. This calls into question the validity of the different models, and creates a need for further research on the nature of the S and L alleles related to this polymorphism so as to correctly identify which model is accurate, or if a different model is needed.

The studies that included an epigenetic component examine it through DNA obtained from blood, rather than from the brain (see Table 5), which call into question the accuracy to which the epigenetic results reflect central brain processes. That said, it is often unethical to use more invasive research methods, and there is increasing evidence that the use of peripheral biomarkers to assess psychopathological disorders provides an acceptable proxy for neurobiological processes, especially in relation to epigenetics [50]. As well, research has recognised a correspondence between brain-derived and blood-derived gene expression signals, indicating that epigenetic patterns observed through peripheral biomarkers are likely an acceptable indication for epigenetic patterns occurring in the brain [32, 34, 50].

Finally, most studies (*n* = 3) used BN and BSD interchangeable. Although they do have different diagnostic criteria, evidence suggests that BSD does not significantly differ from BN on many clinical variables [3]. Therefore, this may be acceptable; however, the results are not specific to either BN or BSD.

## Discussion

The present review identified and appraised seven studies relevant to BN/BSD, BPD, and a genetic/epigenetic component. All the studies indicated differences between individuals with BN/BSD-BPD compared to those with BN/BSD, and HC. When interpreting the findings, it is important to keep in mind the small sample size and nature of the studies included in the review. The current research ultimately suggests that BN/BSD-BPD is likely a different condition from both BN/BSD and BPD; however, much more research is needed to confirm this and to clearly understand the aetiology of the condition.

**Fig. 2:**
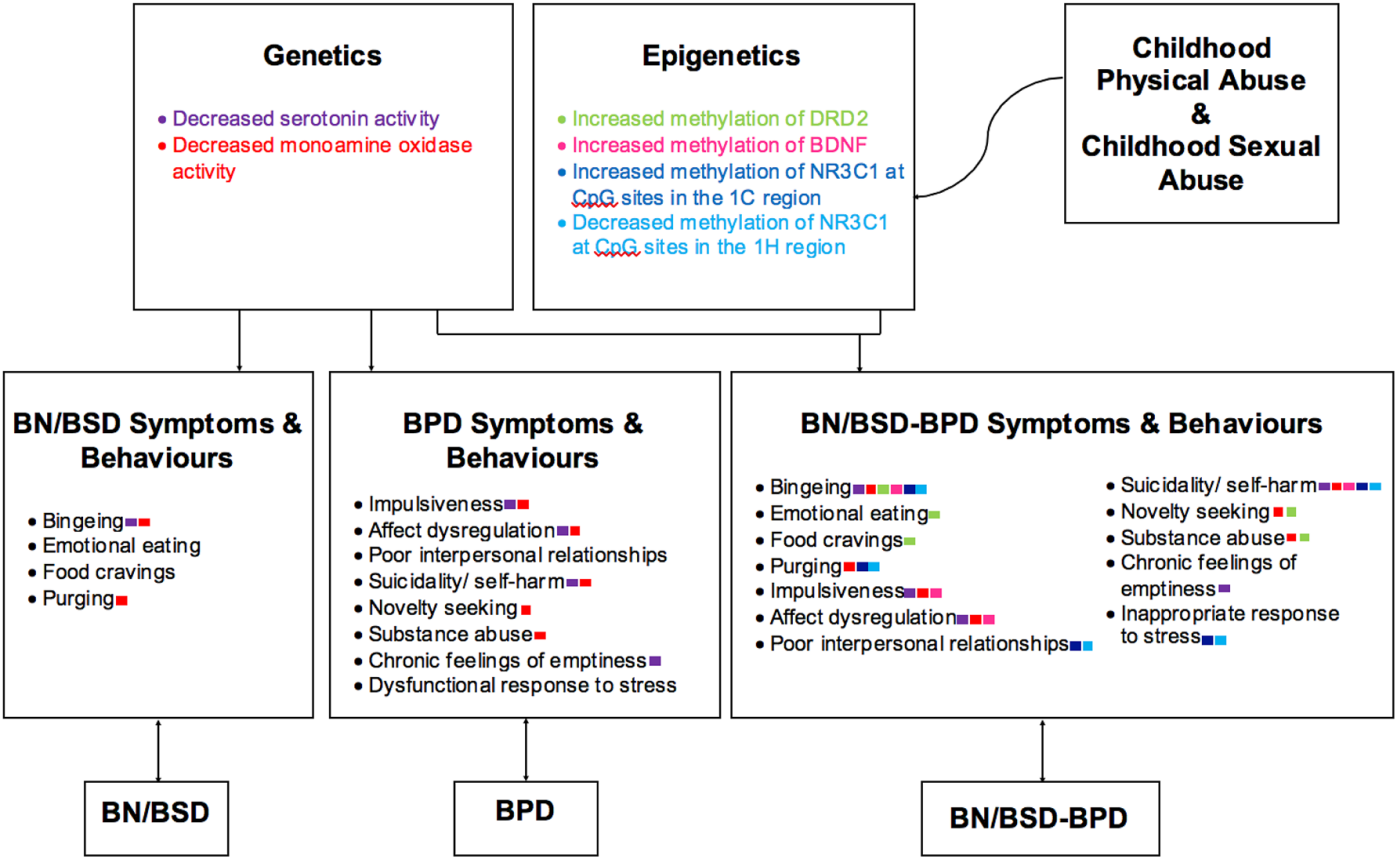
A genetic/epigenetic aetiological model of BN/BSD-BPD. The coloured boxes beside the symptom/behaviour indicate which genetic and/or epigenetic variable(s) are correlated with it

An evidence-based genetic/epigenetic aetiological model of BN/BSD-BPD will be proposed. It is hoped that this model will not only clearly illustrate the current understanding of the genetics/epigenetics relevant to this psychopathology, but will also demonstrate that BN/BSD-BPD is likely different from BN/BSD or BPD (as the current research findings suggest). Generally, the genetic/epigenetic aetiological model of BN/BSD-BPD can be divided into three parts: genetics, epigenetics, and environmental pressures (childhood abuse), where the environmental pressures are largely correlated with the epigenetic effects (see Fig. 2). As the key findings of the papers included in this review have illustrated, individuals with BN/BSD-BPD seem to have genetic differences compared to individuals with BN/BSD or HC as they generally display greater frequencies of the “low-functioning” S allele for the 5-HTTLPR gene polymorphism [20, 24] and largely show reduced MAO activity [28, 43]. These individuals also exhibit gene methylation patterns that are significantly different from individuals with BN/BSD and HC. These methylation differences include changes in methylation of the DRD2 gene, BDNF, and the NR3C1 gene. Further, current research suggests individuals with BN/BSD-BPD were more likely to have experienced childhood abuse (physical and sexual) compared to their counterparts with BN/BSD or HC, and childhood abuse (physical and sexual) was frequently (but not always) associated with the methylation differences observed in this population. Therefore, individuals with BN/BSD-BPD seem to differ from those with BN/BSD and HC on genetic, epigenetic, and environmental levels, suggesting BN/BSD-BPD may be a unique condition. Decreased serotonin and MAO activity is seen in those with BN/BSD and BPD, however, to a lesser degree than seen in BN/BSD-BPD. Serotonin and MAO variation is an important genetic aspect of BN/BSD-BPD, however, epigenetic changes, largely resulting from childhood abuse, seems to be the primary factor that sets BN/BSD-BPD apart from both BN/BSD and BPD as these epigenetic changes are observed to be significantly different when comparing individuals with BN/BSD-BPD to those with BN/BSD or BPD.

When interpreting the genetic/epigenetic aetiological model of BN/BSD-BPD it is important to understand the limitations of the model. Firstly, the model is most relevant to women with BN/BSD-BPD as current literature about BN/BSD-BPD has been conducted exclusively on women. Secondly, only a couple of papers have been published regarding each of the genetic/epigenetic aspects of this condition. Although the findings are convincing when considered alongside previous literature about the relevant genetics/epigenetics, BN/BSD, and independent BPD, more studies are needed to properly validate the findings. The same holds true for the studies about the epigenetic changes. As well, it is important to be aware that the present model almost certainly does not encompass all the genetic and epigenetic differences the BN/BSD-BPD population displays, due to a lack of research on this topic. Only a few studies have been conducted regarding the genetic/epigenetic aspect of BN/BSD-BPD as this condition is just becoming recognized and the research about genetics and epigenetics pertaining to mental health disorders is in its infancy. Furthermore, genotype and DNA methylation also interact for many genes, resulting in potentially different effects arising due to epigenetic changes in some individuals compared to others [51], however, genotype-DNA methylation interactions of the genes under question are not yet clearly understood. Ultimately, the present genetic/epigenetic aetiological model of BN/BSD-BPD serves as a visual representative of the current findings and demonstrates the need for further research on this condition.

It is important to note that the genetic, epigenetic, and environmental factors discussed throughout the present review do not cause an individual to develop BN/BSD-BPD, they simply interact, likely to increase the possibility that an individual may develop this condition. Furthermore, the ways in which these factors interact remains unclear. Recently, an interesting theory was proposed that suggests an individual with a genetic predisposition for a condition may unknowingly seek out environments that could trigger an epigenetic change likely resulting in the emergence of the condition [52]. If this theory holds for the present condition, the genetic/epigenetic aetiological model of BN/BSD-BDP presented above would become more complex and an arrow from the “genetics” box could be added that leads to the “environmental factors” box (childhood sexual and physical abuse), suggesting a more complex interaction between genetics, epigenetics, environmental factors, and psychopathy. Presently, no research has been conducted that specifically examines the genetic predispositions relevant to BN/BSD-BPD (i.e., decreased serotonin and MAO activity) and their influence on an individual’s environmental situations. Future studies that explore this would be of great interest and could be used to make the present genetic/epigenetic aetiological model more complex. As well, these studies would also be beneficial as they would help to determine the degree to which these genetic and epigenetic changes are premorbid or if they are acute state changes associated with BN/BSD-BPD.

## Conclusions

The present systematic review provided an exhaustive summary of the current literature on BN/BSD-BPD and the genetic/epigenetic variables associated with this psychopathology. The results indicated that BN/BSD-BPD is significantly different than BN/BSD and HC on many genetic and epigenetic variables, suggesting that it may be a unique condition. As such, a preliminary genetic/epigenetic aetiological model of this condition was proposed. This model outlines the genetic and epigenetic variables that are currently believed to influence specific symptoms and behaviours of BN/BSD-BPD, and illustrates the likely role of childhood abuse in these conditions.

It is important to acknowledge that the current research is very sparse, and more than anything the present review hopes to illustrate the need for further research regarding the genetic/epigenetic aspects of BN/BSD-BPD. Additional research is needed not only to validate the current findings, but also to more fully understand the genetic/epigenetic aetiology of BN/BSD-BPD. Furthermore, mental health conditions, including BN/BSD and BPD are often comorbid with other psychiatric disorders and research is needed to assess how relevant comorbidities affect the current findings.

## Electronic supplementary material

Below is the link to the electronic supplementary material.

Supplementary material 1 (DOCX 14 kb)
