# Supplementary material for: Understanding the genetics and epigenetics of bulimia nervosa/bulimia spectrum disorder and comorbid borderline personality disorder (BN/BSD-BPD): a systematic review
Source: Eat Weight Disord. 2019 May 22;24(5):799–814. doi: 10.1007/s40519-019-00688-7 (PMC6751148; doi:10.1007/s40519-019-00688-7)
Supplement: Supplementary file 1 — Supplementary material 1 (DOCX 14 kb) [file 40519_2019_688_MOESM1_ESM.docx]

# APPENDIX I: Summary of the search strategy

| Information needed: | Q: What is the relationship between BN, BPD, and genetics and epigenetics?    No limits on publication date, or language | | |
| --- | --- | --- | --- |
| Places to search for information: | Medline, EMBASE, Cochrane Library, PsychINFO, Web of Science, Scopus, CINAHL plus | |  |
| Database searched | Search terms used | Number of Results | Date of search |
| Medline | | | |
| #1 | Bulimia Nervosa/ OR bulimia.mp. | 10085 | 15/11/2018 |
| #2 | Borderline Personality Disorder/ OR borderline adj4 personality disorder*.mp. | 8466 | 15/11/2018 |
| #3 | exp Genes/ OR (gene or genes).mp. OR exp Genetics/ OR genetic*.mp. | 3240852 | 15/11/2018 |
| #4 | 1 AND 2 AND 3 | 10 | 15/11/2018 |
| EMBASE | | | |
| #1 | bulimia/ OR bulimia.mp. | 15025 | 15/11/2018 |
| #2 | borderline state/ OR borderline adj4 personality disorder*.mp. | 13376 | 15/11/2018 |
| #3 | exp genes/ OR (gene or genes).mp. OR exp genetics/ OR genetic*.mp. | 4176288 | 15/11/2018 |
| #4 | 1 AND 2 AND 3 | 24 | 15/11/2018 |
| Cochrane library | | | |
| #1 | bulimia nervosa:kw OR bulimia | 1188 | 15/11/2018 |
| #2 | borderline personality disorder:kw OR borderline near/4 personality disorder* | 803 | 15/11/2018 |
| #3 | Genes:kw OR (gene or genes) OR genetics:kw OR genetic* | 52948 | 15/11/2018 |
| #4 | 1 AND 2 AND 3 | 5 | 15/11/2018 |
| PsychINFO | | | |
| #1 | Bulimia/ OR bulimia.mp. | 11177 | 17/11/2018 |
| #2 | Borderline Personality Disorder/ OR (borderline adj4 personality disorder*).mp. | 20872 | 17/11/2018 |
| #3 | exp genes/ OR (gene or genes).mp. OR exp GENETICS/ OR genetic*.mp. | 144787 | 17/11/2018 |
| #4 | 1 AND 2 AND 3 | 19 | 17/11/2018 |
| Web of Science | | | |
| #1 | Bulimia Nervosa/ OR bulimia.mp. | 13931 | 17/11/2018 |
| #2 | Borderline personality disorder OR borderline near4/ personality disorder* | 12475 | 17/11/2018 |
| #3 | genes OR (gene or genes) OR genetics OR genetic* | 3515772 | 17/11/2018 |
| #4 | 1 AND 2 AND 3 | 21 | 17/11/2018 |
| Scopus | | | |
| #1 | KEY (bulimia AND nervosa) OR ALL (bulimia) | 43192 | 17/11/2018 |
| #2 | KEY (borderline AND personality AND disorder) OR ALL (borderline W/4 personality disorder*) | 44706 | 17/11/2018 |
| #3 | KEY (genes) OR ALL (gene or genes) OR KEY (genetics) OR ALL (genetic*) | 9227839 | 17/11/2018 |
| #4 | 1 AND 2 AND 3 | 983 | 17/11/2018 |
| CINAHL plus | | | |
| #1 | TX bulimia nervosa OR TX bulimia | 4393 | 17/11/2018 |
| #2 | TX borderline personality disorder OR TX borderline n4 personality disorder* | 3146 | 17/11/2018 |
| #3 | TX genes OR TX (gene or genes) OR TX genetics OR TX genetic* | 216041 | 17/11/2018 |
| #4 | 1 AND 2 AND 3 | 4 | 17/11/2018 |
| Total number of studies: | 1066 | | |
| Total number of studies after deduplication: | 1006 | | |
